# Artemether–lumefantrine and dihydroartemisinin–piperaquine treatment outcomes among children infected with uncomplicated *Plasmodium falciparum* malaria in Mwanza, Tanzania

**DOI:** 10.1186/s41182-021-00383-3

**Published:** 2021-11-27

**Authors:** Karol J. Marwa, Eveline T. Konje, Anthony Kapesa, Erasmus Kamugisha, Stanley Mwita, Göte Swedberg

**Affiliations:** 1 Department of Pharmacology, Catholic University of Health and Allied Sciences, Mwanza, Tanzania; 2 Institute of Medical Biochemistry and Microbiology, Uppsala University, Uppsala, Sweden; 3 Department of Epidemiology, Catholic University of Health and Allied Sciences, Mwanza, Tanzania; 4 Department of Community Medicine, Catholic University of Health and Allied Sciences, Mwanza, Tanzania; 5 Department of Biochemistry, Catholic University of Health and Allied Sciences, Mwanza, Tanzania; 6 School of Pharmacy, Catholic University of Health and Allied Sciences, Mwanza, Tanzania

**Keywords:** Efficacy, Treatment outcome, Artemether–lumefantrine, Dihydroartemisinin–piperaquine and Tanzania

## Abstract

**Background:**

Artemisinin based combination therapies (ACTs) have been a cornerstone in the treatment of malaria in the world. A rapid decline in dihydroartemisinin piperaquine (DHP) and artemether lumefantrine (ALU) efficacies has been reported in some parts of South East Asia, the historical epicenter for the antimalarial drug resistance. Prolonged drug use is associated with selection of resistant parasites due to exposure to inadequate drug levels hence effects on treatment outcomes in malaria. ALU and DHP are used as first line and alternative first line, respectively, in Tanzania. This study was carried in Igombe, Tanzania to assess the efficacies of ALU and DHP in routine treatment of uncomplicated malaria among children.

**Methods:**

This was a prospective study involving children up to 10 years and followed up for 28 and 35 days as per the WHO protocol, 2015 for monitoring antimalarial drug efficacy. The primary end points were crude and adjusted Adequate Clinical and Parasitological Response (ACPR), parasite clearance rate and reported adverse events.

**Results:**

A total of 205 children with uncomplicated malaria were enrolled. One hundred and sixteen participants were treated with ALU, while 89 participants were treated with DHP. Two participants in the ALU group were lost within the 24 h of follow-up. The PCR unadjusted ACPR was108 (94.7%) for ALU and 88 (98.9%) for DHP, while the PCR adjusted ACPR was 109(95.6%) and 88(98.9%) for ALU and DHP, respectively, at 28 day follow-up. No treatment failure was observed in both groups. Cumulative risk of recurrent parasitemia was similar in both groups (*p* = 0.32). Age and parasite density were strong predictors for persistent day 1 parasitemia (*p* = 0.034 and 0.026, respectively). Nausea and vomiting, abdominal pain and headache were the most clinical adverse events reported in both groups of patients.

**Conclusion:**

The present study shows that ALU and DHP are still efficacious after more than a decade of use with PCR corrected efficacies greater than 95% implying a failure rate less than 5% which is below the WHO minimum threshold requirement for recommendation of a change in the treatment policy. Both drugs were well tolerated with no major adverse events reported.

## Introduction

The number of malaria cases has declined worldwide by 18% between 2000 and 2015 [1]. However, malaria still remains a public health challenge in Tanzania despite the reported decrease in prevalence by about 10% for the past 10 years. The proportion of children treated with ACT has increased in Sub-Saharan Africa [1] whereby Artemether–lumefantrine (ALU) is the most adopted Artemisinin based Combination Therapy (ACT) for the treatment of uncomplicated *P.falciparum* malaria [2]. Globally, ACT use is estimated to have played part in the decrease in malaria mortality rate by 48% since its introduction to 2015 [1, 3]. Tanzania mainland introduced ALU as the first line drug against uncomplicated malaria in 2006 after the transition from chloroquine (CQ) to Sulphadoxine–pyrimethamine (SP) to artemisinin monotherapy. Resistance has been a driving force for the above transition like in other malaria endemic countries [3–6]. Dihydroartemisinin–piperaquine (DHP) is used as an alternative first line drug in Tanzania. The drug provides longer prophylactic effect from re-infection after treatment up to 65 days due to the longer half-life of the partner drug piperaquine [7]. Piperaquine, a bisquinoline once used in China when chloroquine resistance was high has not been used as monotherapy in any country since 1990s [4, 8]. A rapid decline in DHP and ALU efficacies has been reported in some parts of South East Asia, the historical epicenter for the spread of antimalarial drug resistance [9]. Resistance to older antimalarials used as monotherapies emerged from the SEA and spread to Africa with overwhelming effects [10–12]. Limited update data are available on DHP clinical efficacy in WHO African region including Tanzania. The available data in some parts of the region indicate that DHP has retained high efficacy [13, 14], while in some parts, the drug has recorded inadequate efficacy [15]. No data is available for DHP efficacy and safety in the studied region in Tanzania. Studies on ALU from some parts of Tanzania mainland have reported treatment failure rate below 5% [16–20] which is less than the WHO recommended (10%) for change in the national antimalarial treatment policy [21].

Prolonged drug use is associated with selection of resistant parasites due to exposure to inadequate drug levels hence effects on treatment outcomes in malaria. ALU is the mostly used ACT in Tanzania because it is the first line drug whereas DHP is alternative first line. Therefore, DHP use is expected to be limited than ALU. This study evaluated clinical outcomes of ALU and DHP for the treatment of uncomplicated malaria in Igombe, Tanzania a decade after the introduction of ACTs in the country.

## Methods

### Study area

This was a prospective study conducted from April 2017 to May 2019 at Karume Health Centre in Igombe, a semi-urban and malaria meso-endemic area in Ilemela district, Mwanza region, Tanzania with a population of about 40,000. Igombe is one of the eight sentinel sites established for conducting therapeutic efficacy studies on antimalarial drugs as part of the recommendations of the World Health Organization.

### Sample size determination

The sample size was determined based on the WHO standard protocol for conducting antimalarial efficacy studies [21, 22]. An expected treatment failure rate of 5% was assumed for both drugs used. With 95% confidence interval, 5% precision and 20% loss to follow-up or withdrawal, a minimum sample number of 88 was deemed sufficient for each group of patients.

### Patient recruitment

The data for the study was prospectively collected from children with uncomplicated *P. falciparum* malaria attending the outpatient clinic. Upon seeing the clinician, a full medical history was recorded, followed by a clinical examination as per the Tanzania guideline for management of malaria. Children with signs or symptoms suggestive of uncomplicated malaria were sent to the laboratory where a rapid diagnostic test was done followed with microscopic examination. For those children who were *P. falciparum* positive after laboratory results and met the inclusion criteria as per the WHO protocol for assessment of antimalarial efficacy, a parent or guardian was consulted for the consent to participate in the study after a thorough explanation on the study aims, benefits and rights to reject or withdraw from the study.

Eligible patients were children up to 10 years with fever or history of fever above 37.5 in the past 24 h, microscopically confirmed *P. falciparum* mono-infection (parasite density of 1000–200,000 trophozoites/µL). Children with symptoms of severe malaria according to the WHO case definition, comorbid infection, epilepsy, malnutrition, chronic diseases, history of drug allergy, history of traditional herbs use in the past 4 weeks, any antimalarial drug use in the past 4 weeks, known liver dysfunction or disease and severe anaemia were excluded from this study. Children who had previously been allocated to ALU group were not included in the DHP group once they had malaria episode(s).

### Drug administration and treatment

A clinician prescribed to the children enrolled in the two groups artemether–lumefantrine or dihydroartemisinin–piperaquine which are drugs used as first line and alternative first line, respectively, as per the Tanzania guideline for management of malaria. The first phase involved treatment of one hundred and sixteen children enrolled with artemether lumefantrine (ALU). A follow-up was done for 35 days. When the follow-up for patients on ALU was completed, a switch into treatment using DHP was done then a follow up was done for 35 days. A total of 89 children were treated with dihydroartemisinin–piperaquine (DHP). Artemether 20 mg–lumefantrine 120 mg (Lumartem, Cipla, India) was administered twice daily during three days, while dihydroartemisinin 20 mg—piperaquine 160 mg (Duocotexin® holley-cotec, Beijing) was given once daily as per manufacturer’s dosing schedule based on body weight. The first, third and fifth doses for ALU were administered as direct observation therapy (DOT). Evening/night doses were given at home. A nurse phoned parents and guardians to remind them it was time for taking the medication. To asses for compliance, parents or guardians were requested to bring the empty pill packages in the following morning. Mothers were insisted to breastfeed their children to increase bioavailability of lumefantrine. All doses for DHP were administered as DOT. Children were observed for 1 h after medication to monitor for vomiting. In case of vomiting within 30 min after medication administration, a full repeat dose was given. In the event of persistent vomiting or symptoms, the patient was excluded from the study and received rescue treatment in accordance to the national malaria treatment guideline.

### Follow-up and sample collection

Guardians or parents were requested to return with the children for follow-up. Blood from finger pricks was collected on filter paper (FTA®Whatman paper) on day 0,1,2,3,7,14,21,28,35 for PCR genotyping of Merozoite Surface Protein 1(MSP1) and Merozoite Surface Protein (MSP2) to distinguish between recrudescence and reinfection, MSP1 and MSP2 alleles to establish genetic diversity and allele frequencies (to be published elsewhere) and CYP3A4*1B plus CYP3A5*3 to establish association with plasma concentrations. Venous blood (2mls) was also collected on day 0,1,2,3,7 and 14 for lumefantrine and piperaquine quantification (to be published elsewhere). Thick and thin blood smears were stained by Giemsa (on each day of the visit) according to the WHO standard protocol [23]. Parasite identification and counting were done by two independent experienced microscopists.

### DNA extraction and molecular investigations

Capillary blood sample was collected on Whatman no 1 filter paper during the visits to the health center. The filter papers were then dried at room temperature and kept in sealed plastic bags. Parasite DNA was extracted from the dried blood spots (DBS) on day 0 and the respective days of reappearance of parasites using Life Sciences genomic DNA kit for dried spots according to the manufacture’s protocol. Nested PCR was done to identify MSP1 and MSP2 allele variants using a method described previously [24]. The results were classified as recrudescence or re-infection according to the WHO guideline [25].

### Treatment outcomes

The WHO 2015 protocol [21, 22] was used to classify treatment outcomes as early treatment failure (ETF), late clinical failure (LCF), late parasitological failure (LPF) and adequate clinical and parasitological response (ACPR). Secondary outcomes included parasite clearance rate and occurrence of adverse events. Treatment failures were classified as recrudescence or re-infection after PCR correction.

### Statistical analysis

We used Ms-Excel for data entry and cleaning. All statistical analyses were performed using STATA version 13.1(Statistical Corporation, College Station, TX, US). Categorical data were compared using chi square tests or fisher exact tests where appropriate. Student *t* test was used to compare continuous data for the two groups where necessary. Per-protocol analysis carried, patients who withdrew from the study or were lost to follow-up or had re-infection were not included in the denominator. Kaplan Meier analysis was done to determine the cumulative probability of recurrence-free survival over the 35 day follow-up. Univariate and multivariate analyses of risk factors associated with parasitemia persistence on day 1 were done using generalized linear model with link Poisson due to large variance.

## Results

### Study subjects

Among 602 screened patients,336 patients were malaria positive. Thirty-six children were excluded because they had taken ALU before visiting the clinic and 44 children did not participate in the study because their parents or guardians did not give a consent. We excluded 14 children because they had taken traditional herbs before visiting the clinic and 33 children were excluded because they had severe malaria or mixed-species infections. Four children were excluded because of persistent nausea and vomiting. Therefore, total of 205 children with falciparum malaria were enrolled in the study. Two patients in the ALU group were lost within the 24 h of follow-up hence were not included in the analysis involving treatment outcomes.

In this study that investigated the efficacy, 116 and 89 participants were treated with ALU and DHP, respectively. Pre-treatment characteristics of the study participants are presented in Table 1

**Table 1:** Baseline characteristics of the study participants

Characteristics	ALU	DHP
	N = 116	N = 89
Sex		
Male *n* (%)	58 (50.00%)	42 (47.19%)
Female *n* (%)	58 (50.00%)	47 (52.81%)
Age in years		
Mean (SD)	5.83 (3.06)	5.92 (3.22)
Weight in kg		
Median (IQR)	18.55 (11.30)	18.30 (15.20)
Height in Cm		
Mean (SD)	108.86 (22.60)	110.82 (25.26)
BMI (Kg/m^2^)		
Underweight (< 5th percentile) *n* (%)	5 (4.31)	4 (4.49)
Normal (5th to 85th percentile) *n* (%)	94 (81.03)	72 (80.90)
Overweight (85th–95th percentile) *n* (%)	12 (10.34)	9 (10.11)
Obese (≥ 95th percentile) *n* (%)	5 (4.31)	4 (4.49)
Temperature (^o^C)		
Mean (SD)	37.89 (0.76)	37.78 (0.98)
Hemoglobin (g/dL)		
Mean (SD)	10.48 (1.78)	10.86 (1.41)
RBG (mmol/L)		
Mean (SD)	4.80 (1.42)	5.02 (1.20)
Hepatitis (YES)		
*n* (%)	0	0
Parasite count at Time zero (/µl)		
Geometric mean	15,413.06	10,715.71

### Treatment outcomes

ETFs were not recorded in both groups of patients. The per protocol day 28 PCR corrected efficacies were high for both group of patients. Both drugs recorded lower LCF and LPF (Table 2).

**Table 2:** Crude and adjusted treatment outcomes

Treatment outcome	ALU (N = 114)	DHP (N = 89)
ETF	0 (0%)	0 (0%)
LCF, *n* (%)	1 (0.88%)	0 (0%)
LPF, *n* (%)	5 (4.39%)	1 (1.12%)
ACPR, *n* (%)	108 (94.74%)	88 (98.88%)
PCR corrected		
ETF	0	0
LCF, *n* (%)	1 (0.88)	0
LPF, *n* (%)	4 (3.54)	1 (1.12)
ACPR, *n* (%)	109 (95.58)	88 (98.88)
Parasite persistence		
Positive blood smear		
Day 1, *n* (%)	50 (43.10)	36 (40.45)
Day 2, *n* (%)	3 (2.59)	1 (1.12)
Day 3, *n* (%)	0	0

The percentage of parasite clearance at day1 were about 50% for both groups. All patients in both treatment groups were aparasitemic on day 3 (Table 2).

*ETF* Early Treatment Failure, *LCF* Late Clinical Failure, *LPF* Late Clinical and Parasitological Failure

**Fig. 1:**
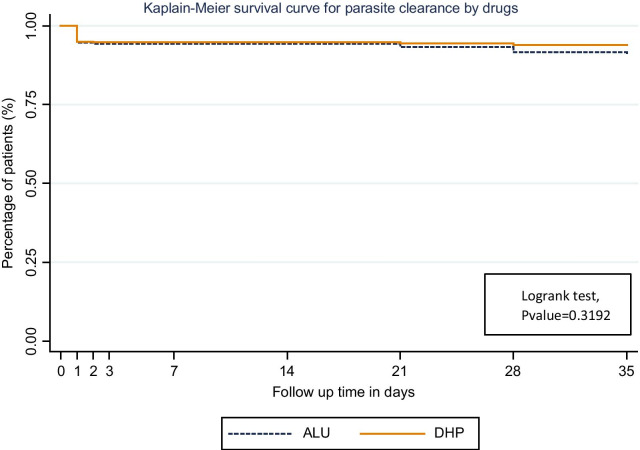
Cumulative risk of recurrent parasitemia by 35 following treatment with artemether–lumefantrine and dihydroartemisinin–piperaquine–Cox-proportional hazards risk

The rate of parasite recurrence during 35 days follow-up was not different in the two treatment groups as suggested in Fig. 1.

**Fig. 2:**
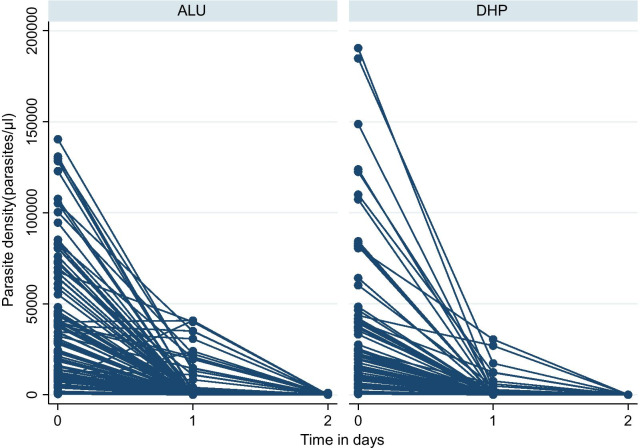
Parasite clearance rate for DHP and ALU

Figure 2 shows the rate of parasite clearance by drugs in day1 and day2. The proportion of the parasite cleared in day1 was 80.44% and 96.41% for ALU and DHP, respectively, while by day 2, both drugs reached 99.99% clearance. In general, there was a rapid parasite clearance in both treatments although parasite clearance time was less in DHP than ALU.

Age and parasite density were strong predictors for persistent day 1 parasitemia. The patients aged 5 years and above were less likely to have parasitemia persistent in day 1 than those under five years. Higher pretreatment temperature (> 37.5), drug type, higher hemoglobin, sex and BMI were not predictors for persistent day 1 and 2 parasitemia (Table 3).

**Table 3:** Association between factors and a positive blood smear in day one

Factor	+ blood smear in day 1	Univariate analysis		Multivariate analysis	
		RR (95% CI)	*p* value	RR (95% CI)	*p* value
Drug					
ALU	43.10%	Ref			
DHP	40.45%	0.94 (0.67–1.30)	0.704		
Sex					
Female	46.67	Ref			
Male	37.00	0.79 (0.57–1.10)	0.166		
Age					
≤ 5 years	50.48	Ref		Ref	
> 5 years	33.00	0.65 (0.47–0.92)	0.014	0.71 (0.52–0.97)	0.034
RBG (mmol/L)					
< 5	39.00	Ref			
≥ 5	41.05	1.05 (0.75–1.49)	0.770		
Hb g/dL					
≤ 10	44.62	Ref			
> 10	38.06	0.86 (0.60–1.21)	0.370		
BMI (kg/m^2^)					
Normal	42.68	Ref			
Underweight	45.45	1.07 (0.54–2.09)	0.855		
Overweight	40.00	0.94 (0.53–1.65)	0.822		
Obese	30.00	0.70 (0.27–1.85)	0.474		
Parasite density (parasite/µl)					
Min–3999	12.77	Ref		Ref	
4000–20,000	30.36	2.38 (1.02–5.55)	0.045	2.64 (1.12–6.21)	0.026
20,001–Max	61.76	4.84 (2.25–10.39)	0.000	4.75 (2.20–10.25)	0.000
Temp. in °C					
< 37.0	26.32	Ref		Ref	
37.0–37.9	47.83	1.82 (1.01–3.27)	0.046	1.62 (0.95–2.78)	0.077
≥ 38.0	42.71	1.62 (0.91–2.90)	0.103	1.32 (0.05–0.24)	0.318

**Adverse events** Nausea and vomiting, abdominal pain and headache were the most clinical adverse events reported in both groups of patients. (Table 4).

**Table 4:** Patient-reported adverse events

Adverse effect	ALU		DHP	
	Patient reported	*N* (%)	Patient reported	*N* (%)
Headache (yes) *n* (%)	98	12 (12.24%)	88	8 (9.09%)
Nausea and vomiting (yes) *n* (%)	99	14 (14.14%)	87	3 (3.45%)
Cough (yes) *n* (%)	99	4 (4.04%)	88	0
Diarrhea (yes) *n* (%)	98	1 (1.02%)	88	3 (3.41%)
Loss appetite (yes) *n* (%)	93	5 (5.38%)	89	1 (1.12%)
Abdominal pain (yes) *n* (%)	98	13 (13.27%)	86	7 (8.14%)

## Discussion

The present study aimed at assessing the efficacy of ALU and DHP in routine treatment of uncomplicated malaria among children after more than a decade since the introduction of ACTs in Tanzania [26].

The per protocol day 28 PCR corrected efficacies were higher for both DHP and ALU. Several studies in other parts of Africa have reported high efficacy for DHP than ALU. Both drugs have shown higher efficacy than the cutoff value recommended by WHO for a change in the national antimalarial treatment policy [21, 22] similar to studies done in other parts of the country [16–20]. In general, this study shows that the two ACTs have retained high efficacies after about 14 years since the introduction of ACTs. Previous studies in the country have suggested the selection of Pfmdr1 N86 and PfcrtK76 does not affect the efficacy of ALU [20, 27]. Validated K13 mutants responsible for artemisinin resistance thus affecting treatment outcome have not been reported in the country [16, 17]. The contribution of the partner drug piperaquine to the parasite killing effect soon after drug administration may account for the high DHP efficacy observed [28]. Piperaquine has a longer half-life (2–3 weeks) than lumefantrine (4.5 days) which may also explain the lack of re-infection observed with DHP. In Tanzania, DHP is deployed as alternative first line treatment and its use is limited owing to the high cost as the drug is not subsidized unlike ALU. This may account for less drug pressure on *P. falciparum* and hence the high efficacy retained. However, experience from the Great Sub-Mekong region has shown a rapid decrease from the high DHP efficacy as a result of a rapid resistance selection to piperaquine [9, 29] possibly due to the long half-life of piperaquine thus warning us on the high efficacy we are enjoying now. This calls for close monitoring for DHP resistance.

Aparasitemia on day 3 in both treatment groups suggests an adequate response of *P. falciparum* to the artemisinin component and an indication of absence of artemisinin resistance in the study area as per the WHO’s indicator of day 3 parasitemia ≥ 10% [30]. Several factors related to drug, host and parasite determine the speed of parasite clearance [31–33]. The effect observed with age on persistent day 1 parasitemia in this study could be attributed to less acquired natural immunity among under-fives in the malaria endemic region. The present study also documents low rates of parasite recurrence for the two drugs (ALU and DHP) used for the treatment of *P. falciparum* malaria in Tanzania.

This study reports both ALU and DHP to be very well tolerated with comparable profiles. No patient experienced serious adverse event in both treatment groups during the follow-up whereby nausea and vomiting were observed more frequently in patients on ALU than DHP. This is not surprising since several studies have reported nausea and vomiting as the most common adverse event associated with ALU use [34]. The high tolerability observed in both drugs is consistent with other studies [16, 35]. The assessment of adverse events in malaria treated patients is complex because of the high background malaria signs and symptoms. To take care of this, clinical assessment was done prior administration of the ACTs. We only considered those events which occurred post start of treatment or worsened after the start of treatment as reported in previous studies [36].

Our study has some limitations. First, determination of the gametocytes clearance time was not done in both groups of patients. Secondly, molecular makers of resistance for both drugs were not studies; therefore, the association between these makers and recurrence of parasites was not done. The pill count method used to assess compliance of the night doses for artemether lumefantrine has some limitations, thus it could not be verified with certainty all children received the night doses which were not under DOT. However, previous studies done in similar settings indicated a high adherence to ALU under real-life situation and the non adherence was due to untimeliness rather than missing doses[37, 38]. The efficacy for the two drugs was based on followup up to 28 and 35 days only. Despite the limitations, our study provides an update on the efficacies of the ALU and DHP in the country.

## Conclusion

ALU and DHP have retained a high efficacy in the treatment of uncomplicated *plasmodium falciparum* malaria. Age and parasite density were strong predictors for persistent day 1 parasitemia. The use of both ALU and DHP was associated with tolerable adverse effects.

## Abbreviations

ACT: Artemisinin-based combination therapy
DHP: Dihydroartemisinin–piperaquine
ALU: Artemether–lumefantrine
ACPR: Adequate clinical and parasitological response
ETF: Early treatment failure
LCF: Late clinical failure
LPF: Late parasitological failure
WHO: World Health Organization
